# Periodontal, salivary and IL-6 status in rheumatoid arthritis patients. A cross-sectional study

**DOI:** 10.4317/medoral.21937

**Published:** 2017-08-16

**Authors:** Javier Silvestre-Rangil, Leticia Bagán, Francisco-Javier Silvestre, Mayte Martinez-Herrera, José-Vicente Bagán

**Affiliations:** 1 Associate profesor. Department of Stomatology. University of Valencia, Spain; 2 University of Valencia. Medical and Dental School. Spain; 3 Assistant Professor of Stomatology. Faculty of Medicine and Odontology. University of Valencia, Spain; 4 University of Valencia, Spain; 5 Professor of Oral Medicine. Valencia University Medical and Dental School. Chairman Service of Stomatology and Maxillofacial Surgery, University General Hospital, Valencia, Spain

## Abstract

**Background:**

The aim of this study was to determine whether saliva interleukin-6 (IL-6) levels are elevated in patients with rheumatoid arthritis versus a control group and examine the possible relationship between the oral condition and the risk of RA.

**Material and Methods:**

In 30 patients with RA and 30 healthy controls, different periodontal indices were recorded; sialometric measurements were taken to determine resting whole saliva, stimulated whole saliva and stimulated parotid saliva flow; and the saliva IL-6 levels were measured. Logistic regression analysis was performed, with the presence or absence of RA as dependent variable.

**Results:**

The patients with RA had a greater presence of bacterial plaque, a greater periodontal pocket depth, a larger percentage of medium-sized pockets, and greater periodontal attachment loss compared with the controls. Likewise, a decrease in resting and stimulated saliva flow was observed, together with an increase in saliva IL-6 levels. Logistic regression analysis reported that the plaque index is the principal differentiating factor of patients with RA. Stimulated parotid saliva flow was also significantly correlated to the presence of RA.

**Conclusions:**

The patients with RA showed a greater tendency to develop periodontal disease than the controls, with lower salivary flow and higher levels of IL-6 in saliva.

** Key words:**Rheumatoid arthritis, periodontal disease, saliva, IL-6.

## Introduction

There is scientific evidence of an association between oral inflammatory disorders and certain systemic diseases (1). Specifically, periodontal disease (PD) has been related to a number of systemic inflammatory disorders (2), including rheumatoid arthritis (RA) (1,3,4-8). Patients with RA appear to suffer a significantly increased incidence of alveolar bone loss (3,5-8), and more periodontal problems are seen in patients with severe and advanced RA (8). It has also been postulated that patients with RA have greater difficulties in maintaining an adequate oral hygiene (9), though this could only partially explain the relationship between the two diseases (10).

Both RA and PD are complex and multifactorial disorders, and have a number of characteristics in common (8,11). The main difference between them is the fact that RA is an autoimmune disease, characterized by a specific adaptive immune response to the presence of an autoantigen. The resulting inflammation is prolonged over time, and eventually develops joint tissue destruction (11). Inflammation is also a fundamental feature of PD, though in this case it is a consequence of the bacterial plaque (11).

The literature contains many studies suggesting similarities between RA and periodontitis (7,12,13). In this context, it has been reported that inflammation in PD results in soft tissue and alveolar bone destruction similar to that seen in the joint tissues of patients with RA (13). Furthermore, both processes are characterized by an exaggerated inflammatory response regulated by the infiltration of immune cells, enzymes and cytokines (13).

Porphyromona gingivalis, as well as smoking, have been suggested as possible factors intervening in RA (14). Both are able to induce the production of citrullinated proteins, which in susceptible individuals could result in anti-citrullinated protein antibody production. This in turn could give rise to osteoarthritis by triggering an immune response at joint synovial membrane level (14-16).

Cytokines, including interleukins, are soluble proteins that play an important role in the initiation and maintenance of inflamma-tory and immune responses, and in intercellular communication (17). As a result, they have been investigated in the context of diseases such as those dealt with in our study. It has been suggested that interleukin-6 (IL-6) plays an important role in the relationship between RA and PD (18,19). Specifically, it has been postulated that individual variability in the capacity to synthesize IL-6 can modulate the susceptibility, development and progression of inflammatory and autoimmune disorders such as RA (17,20). Furthermore, the inhibition of this interleukin has been shown to exert beneficial effects in the treatment of RA (17,21). Other cytokines such as interleukin-1 (IL-1) or tumor necrosis factor-alpha (TNF-α) may also participate in the relationship between RA and PD.

The objectives of this study were to determine the periodontal and salivary variables most related to the risk of RA, and even examine wether interleukin-6 (IL-6) levels are elevated in patients with rheumatoid arthritis versus a control group.

## Material and Methods

A randomized, prospective case-control study was made of 30 patients with RA treated and followed-up on in the Department of Rheumatology (Dr. Peset University Hospital, Valencia, Spain) (Group 1), and 30 age- and gender-matched controls randomly selected from among the patients seen in the Dental Clinic (University of Valencia, Valencia, Spain) (Group 2). All patients were reviewed between January 2010 and January 2013. The study was approved by the Ethics Committee of Dr. Peset University Hospital, and all patients gave informed consent to participation.

As inclusion criteria, the patients in group 1 were required to have a diagnosis of RA, with follow-up of the disease; RA was required to be the only autoimmune disorder; and teeth were required to be present in the mouth. Patients with additional systemic diseases capable of presenting inflammatory activity were excluded from the study. In the control group we excluded those individuals with known systemic disorders. Lastly, pregnant or breastfeeding women were excluded in both groups, together with patients subjected to antibiotic treatment in the last month or periodontal treatment in the last 6 months.

The mean age in the group of patients with RA was 60.7 ± 7,6 years, versus 58.6 ± 6.7 years in the control group (t=0.276; *p*>0.05). In turn, in both groups 8 of the patients were males (26.66%) and 22 were females (73.33%) (χ2= 1; *p*>0.05).

With regard to the explorations and tests, the subjects in both groups were required to report under fasting conditions at between 8-10 in the morning, and were instructed not to smoke or brush their teeth before the visit. The Silness and Löe plaque index was recorded in all patients (22). We measured periodontal pocket depth at 6 points per tooth using a WHO probe, as well as gingival recession, and calculated periodontal attachment loss. The percentages of physiological (1-3 mm), moderate (4-5 mm) and severe (> 6 mm) pockets were measured in each patient. Bleeding in response to probing was recorded. Lastly, sialometry was used to determine resting whole saliva (RWS), stimulated whole saliva (SWS) and stimulated parotid saliva flow (SPS) (23). The RWS and SPS measurements were made using the expectoration technique with the collection of saliva during 5 minutes. Secretion was stimulated by chewing a piece of paraffin, and in the case of the SWS measurements, the saliva produced in the first two minutes was discarded. Lastly, for recording the SPS measurements, we placed a Lashley capsule at the opening of the parotid duct during 5 minutes. Saliva production was stimulated with 2% citric acid solution (23). After collecting the saliva and measuring the saliva flow values, the samples were frozen at -80ºC for posterior analysis of the IL-6 levels.

The saliva IL-6 levels were determined using an ELISA kit (Quantikine Human IL-6, R&D Systems, Inc., Minneapolis, MN, USA), according to the instructions of the manufacturer. The saliva samples and standards and controls of the kit were loaded onto the specific anti-IL-6 monoclonal antibody affixed to the plates, followed by incubation for two hours. Washing buffer was used to remove the unbound substances, and specific enzyme-bound anti-IL-6 polyclonal antibody was added to all the wells, followed by incubation for another two hours. After the second washing, the substrate solution was added, followed by incubation during 30 minutes in the dark. The reactions were stopped with sulfuric acid. Absorbance was measured with a spectrophotometer at a wavelength of 450 nm. The IL-6 levels were expressed as the mean ± standard deviation and in pg/ml (24).

Prior to data analysis, we used the Levene test to check the homogeneity of the two groups. A descriptive study was made of the variables, with application of the Student t-test for the evaluation of differences in the means of the variables between the groups, in the presence of a normal distribution. The Mann-Whitney U-test was used in the case of a non-normal sample distribution. The chi-squared test was applied for the comparison of categorical variables.

After identifying those factors exhibiting statistical significance or near significance, a logistic regression model was developed with the presence or absence of RA as dependent variable. The Wald backwards stepwise elimination test was used, and the Nagelkerke R2 coefficient and Hosmer-Lemeshow test were employed for assessing the goodness of fit. Statistical significance was considered for *p*<0.05.

## Results

The comparison of the study variables between the two groups is shown in  Table 1. Bacterial plaque was found to be significantly more prevalent in the patients with RA than in the healthy controls (*p*<0.001).

**Table 1:**
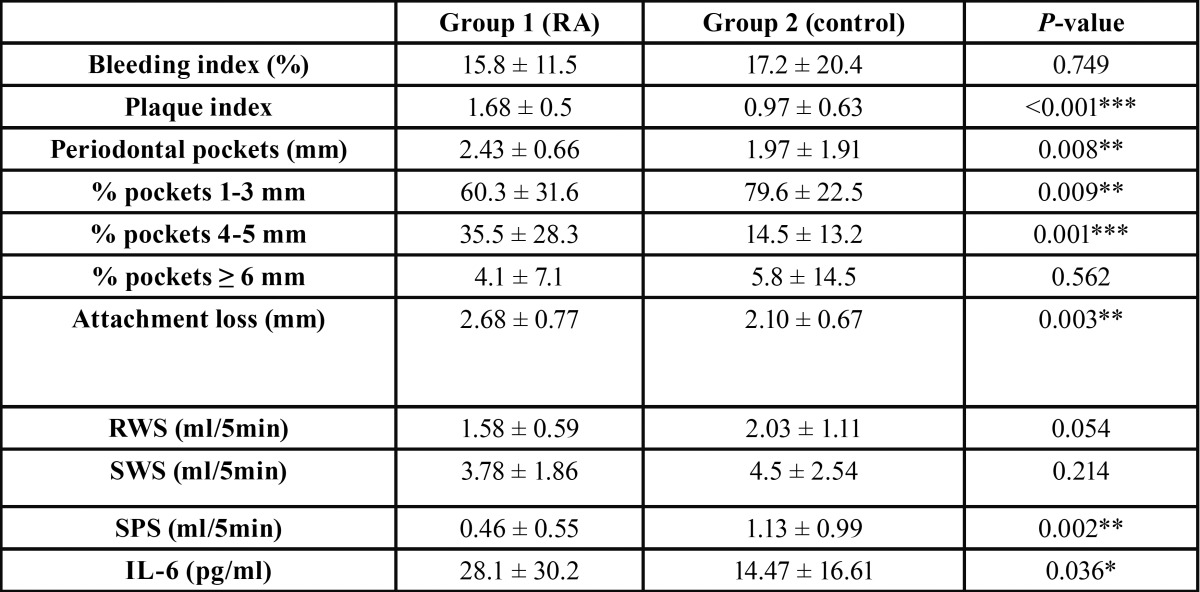
Results of the study parameters in both groups. RWS: resting whole saliva; SWS: stimulated whole saliva; SPS: stimulated parotid saliva; IL-6: interleukin-6.

Regarding the periodontal condition of the patients, the pockets in group 1 were significantly deeper than in the control group (*p*<0.01), and periodontal attachment loss was likewise greater among the patients with RA (*p*<0.01) ( Table 1). Medium pocket depths (i.e., 4-5 mm) were much more prevalent in group 1.

The mean RWS and SPS values were clearly reduced in the patients with RA, though important statistical significance was only reached in the case of SPS (*p*=0.002). Nevertheless, in the case of RWS the statistical analysis revealed a strong tendency among patients with RA to produce less saliva (*p*=0.05).

There were no statistically significant differences between the two groups in terms of bleeding in response to probing (*p*>0.05).

The saliva IL-6 levels were significantly higher in the patients with RA than in the controls (*p*<0.05). However, the logistic re-gression analysis found the plaque index to be the main differentiating factor between patients with RA and the controls. Specifically, for each unit increase in plaque index the risk of suffering RA increased almost 7-fold (OR = 6.77, 95%CI: 1.22-37.55; *p*=0.03) ( Table 2).

**Table 2:**
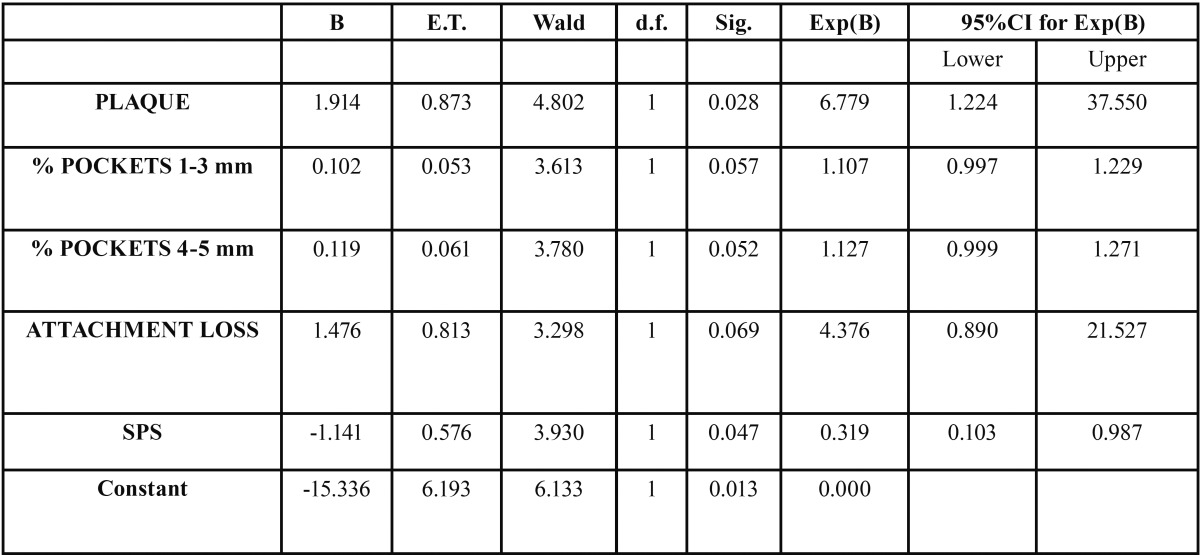
Estimation of coefficients for the logistic regression model referred to the presence or absence of rheumatoid arthritis. Estimation of coefficients for the logistic regression model referred to the presence or absence of rheumatoid arthritis.

The logistic regression analysis also found SPS to explain the presence of RA. In effect, each unit increase in SPS value was associated to a 68% decrease in the risk of RA (OR = 0.32, 95%CI: 0.10-0.99; *p*=0.05)( Table 2).

In contrast, the rest of the periodontal indices, such as pocket depth or periodontal attachment loss were not found to be significantly associated to an increased risk of RA in the logistic regression analysis (*p*>0.05), though the values nevertheless came close to significance ( Table 2).

Lastly, the saliva IL-6 levels likewise showed no statistically significant correlation to the risk of RA in the logistic regression study ( Table 2).

The Hosmer-Lemeshow test confirmed that the model adjusted favorably to the data (*p*=0.84), and the Nagelkerke R2 coefficient was 0.592, which suggests that 59.2% of the variance in the presence of the disease could be explained by the considered parameters.

## Discussion

The first objective of our study was to determine which periodontal and salivary variables are more related to the risk of RA. In a first univariate association, we found that were altered periodontal parameters in patients with RA, which conditioned a greater predisposition of these patients to PD. In this regard, comparison of these parameters between group 1 and the controls showed bacterial plaque to be significantly increased in patients with RA versus the controls (*p* < 0.05). This finding is consistent with the observations of other authors such as Pischon *et al.* 10 and Susanto *et al.* (25). Over time, the accumulation of plaque in patients with RA could lead to the development of periodontal disease. The increase in bacterial plaque in group 1 could perhaps be explained by difficulties in maintaining adequate oral hygiene, due to joint functional limitation and possible deformity.

In agreement with the literature, the recorded increase in mean pocket depth and the observed periodontal attachment loss indicate that patients with RA are more susceptible to the development of periodontal disease (10). Furthermore, the patients without RA had a greater proportion of physiological pockets (1-3 mm), while the RA group was characterized by a greater presence of medium-depth pockets (4-5 mm) indicative of chronic periodontal inflammatory irritation ( Table 1) (15,26).

Salivary function was also found to be altered in the patients with RA. This was particularly notorious in the case of the parotid glands, where a very clear decrease in saliva flow was observed. This phenomenon has been widely studied in patients with Sjögren’s syndrome, though less information is available in the case of patients with RA in the absence of Sjögren’s syndrome. Although the literature is not very extensive regarding the salivary function of patients with RA who don´t present Sjögren´s syndrome, some authors who have measured salivary function in a similar way to ours using sialometries, have obtained similar results to our study (27).

One of our objectives was to develop a logistic regression model capable of explaining the greater or lesser risk of RA based on the saliva and periodontal parameters of the patients, and the evaluation of saliva IL-6 levels.

We found bacterial plaque and SPS to be the clinical parameters with the strongest association to the development of RA. Specifically, the logistic regression analysis identified the plaque index as the main differentiating factor between patients with RA and the controls, with an almost 7-fold increase in the risk of RA for each unit increase in plaque index. In turn, each unit increase in SPS value was associated to a 68% decrease in the risk of RA, indicating a close relationship between RA and bacterial plaque and the amount of parotid saliva.

The logistic regression model also found the presence of medium-depth periodontal pockets to increase the risk of RA. This would be consistent with the increase in bacterial plaque and decrease in resting saliva flow. Lastly, the model identified periodontal attachment loss as a potential risk factor for the development of RA, though in the same way as with pocket depth, the association fell a little short of statistical significance.

Other authors have reported an increased risk of RA in patients with periodontal disease (5,28), and the reverse association has also been observed, i.e., patients with RA have been reported to be at an increased risk of developing periodontal disease (3,10). The data found in the literature, and our own observations, suggest that there may be an association between RA and periodontal disease. The idea that this relationship could be bidirectional as commented above (i.e., RA might influence the risk of periodontal disease, and the latter in turn could contribute to the future development of RA) requires the conduction of longitudinal studies with larger patient samples, rather than preliminary case-control studies such as our own. Indeed, the design involved has been the main limiting factor of our study in attempting to account for a possible relationship between RA and periodontal disease.

According to the “double-hit” periodontitis model, a first “hit” by inflammation produced by periodontitis would be followed by a second “hit” at joint level, with exacerbation of the synovial inflammatory response (12). Another hypothesis suggests the development of an immune response generated by proteins altered by enzymes of periodontal bacterial origin (Porphyromona gingivalis) – giving rise to anti-citrullinated protein antibodies with effects at synovial level (14).

Interleukin-6 is a cytokine with multiple essential functions in immune regulation and inflammatory response. It is mainly produced by T lymphocytes, but is also secreted by macrophages, endothelial cells and fibroblasts (17). The latter appear to be the most abundant source of IL-6 in the inflamed synovial membrane. Interleukin-6 is generally produced in response to nonspecific stimuli such as bacterial factors or other cytokines. Although it is produced locally, it may exert systemic effects by spreading from the inflammatory focus through the bloodstream (17). In addition, IL-6 may be a key mediator in the development of many chronic inflammatory diseases, including RA, where it could play a role in the etiopathogenesis of the disorder by regulating the differentiation and activation of CD4-positive T cells, and intervening in local inflammation (28).

The results of our study show a clear increase in saliva IL-6 levels compared with the healthy controls. The main source of this increase could be crevicular fluid, due to the greater incidence of periodontal disease observed in these patients (26). Costa *et al.* recorded higher saliva IL-6 levels in patients with active periodontitis using a saliva collection technique identical to our own. Likewise, it has been seen that the IL-6 levels decrease after periodontal treatment in patients with periodontitis (29).

On the other hand, the increase in IL-6 levels could also have a systemic origin from the inflamed synovial membrane. Clinical trials with tocilizumab (a monoclonal antibody capable of neutralizing the biological effect of IL-6 by blocking its specific receptor [IL-6R]) have shown that this antibody inhibits the systemic response, with a rapid decrease in the concentrations of C-reactive protein and of other biological parameters dependent upon IL-6 (30).

The use of IL-6 levels in saliva as a marker of RA activity, could be a less invasive test than the constant blood analyses that are subjected to these patients. Therefore, it would seem interesting to carry out studies correlating IL-6 levels in blood and saliva in order to use IL-6 level in saliva as a less invasive marker of the disease.
